# Age-related Impairment of Vascular Structure and Functions

**DOI:** 10.14336/AD.2017.0430

**Published:** 2017-10-01

**Authors:** Xianglai Xu, Brian Wang, Changhong Ren, Jiangnan Hu, David A. Greenberg, Tianxiang Chen, Liping Xie, Kunlin Jin

**Affiliations:** ^1^Zhongshan Hospital, Fudan University, Shanghai 200032, China.; ^2^Department of Pharmacology and Neuroscience, University of North Texas Health Science Center at Fort Worth, TX 76107, USA.; ^3^Department of Urology, the First Affiliated Hospital, Zhejiang University, Zhejiang Province, China.; ^4^Institute of Hypoxia Medicine, Xuanwu Hospital, Capital Medical University. Beijing, China.; ^5^Buck Institute for Research on Aging, Novato, CA 94945, USA.; ^6^Department of Thoracic Surgery, Shanghai Chest Hospital, Shanghai Jiaotong University, Shanghai, China.

**Keywords:** vascular aging, vascular remodeling, endothelial dysfunction, arterial stiffness

## Abstract

Among age-related diseases, cardiovascular and cerebrovascular diseases are major causes of death. Vascular dysfunction is a key characteristic of these diseases wherein age is an independent and essential risk factor. The present work will review morphological alterations of aging vessels in-depth, which includes the discussion of age-related microvessel loss and changes to vasculature involving the capillary basement membrane, intima, media, and adventitia as well as the accompanying vascular dysfunctions arising from these alterations.

The aging process is congruent with the passing of time; it waits for no man. Statistics from the World Health Organization (2012) show that the number of people over the age of 60 has doubled since 1980 and will increase approximately fourfold by the year 2050 [1]. Physiological and pathological aging increases the risk of acute and chronic clinical diseases, leads to adverse outcomes and imposes personal and societal healthcare costs [2].

Vascular dysfunction is a key characteristic of cardiovascular, cerebrovascular and neurodegenerative diseases and aging leads to an impairment of blood vessel function [3]. Although many other risk factors such as cigarette smoking [4] have been reported to decline in vascular function, aging has been considered an independent and essential risk factor. Hence, complicated changes of structure and function of the vasculature have been linked to aging and age-related diseases.

This in-depth review examines structural changes in aging vessels and how these can lead to arterial stiffness, endothelial dysfunction, hypoperfusion, and even blood-brain-barrier dysfunction.

## 1. Macrostructural and microstructural remodeling in vascular aging

### 1.1 Macrostructural alterations in vascular aging

#### Age-related microvessel loss

The preponderance of studies shows that cerebrovascular density declines during normal aging. A published review in 1996 summarized 7 aging studies that revealed mixed results [5]. However, another review published in 2003 showed that the majority of 22 cited papers reported lower vascular densities with aging in both humans and rats [6].

In studies of rodents, most have focused on vascular density and length in the cerebral cortex. Vascular density decreased with age in the cerebral cortex including the frontal, occipital and parietal cortices in Wistar, Wistar-Kyoto (WKY), spontaneously hypertensive (SHR), Brown-Norway, and F344 rats [7-11]. An age-related decrease in the number of venules and arteriole-to-arteriole anastomoses was also reported [12]. Other brain regions show a similar pattern. In the hippocampus and brain stem, capillary density decreased with increasing age in Wistar and F344 rats [13-17]. Moreover, intercapillary distance increased with age in both the hippocampus and cerebral cortex. Capillaries of the young adult rat brain were observed to be clear, interconnected tubular structures while the capillaries of the aged rats were fragmented and disconnected [18]. In the olfactory bulb (OB) and the medial nucleus of the trapezoid body (MTB), which is the largest cell group of the rat superior olivary complex, vascularity decreases with age by 15% and 30%, respectively [15, 19, 20]. In white matter, capillary length and volume were reduced with age [21].

Recently, 3-D analysis methods have been used to assess the capillary profiles in the aged animal. Brain capillary density and branching point were gauged through a detailed 3-D analysis for the purpose of evaluating if there are distinctive regional alterations with advancing age. Capillaries showed significant loss in the cortex, corpus callosum/white matter and hippocampal regions of aged mice with region to region differences in the cortex (19.26 ± 6.7%), corpus callosum/white matter (34.5 ± 10.86%) and hippocampus (26.38 ± 5.63%). Meanwhile, the number of capillary branch points was decreased in the cortex and corpus callosum/white matter with aging while the hippocampus was spared [22]. Two-photon microscopy showed that capillary hematocrit and density were substantially lower in the aged rat group (11 to 15-week-old *vs.* 23 to 25-month-old) by 32% and 20%, respectively [23].

Capillaries in the cerebral cortex of different age groups have also been analyzed in humans. Decreased microvascular density was found in aged individuals in the frontal and calcarine cortices, paraventricular nucleus, hippocampus and superficial and deep white matter, which was not correlated with hypertension [24-26]. In contrast, no change was found in the temporal cortex and supraoptic nuclei. Not every region of the human brain has been assessed; the methods most widely used, immunohistochemistry and light microscopy, are limited in the quality of results.

Magnetic resonance angiograms (MRAs) were also used to examine vessel segmentation in 100 healthy volunteers, from 18 to74 years, without any hypertension or other diseases that may affect the vasculature. Both males and females exhibited a trend toward vessel loss during aging in 4 anatomical regions: left and right middle, anterior and posterior cerebral artery territories. The vessel count within the left middle cerebral artery circulation was significantly lower in subjects age 60 and over *vs* those in the age 18 to 29 group. The most remarkable results were in the posterior circulation, where vessel number was significantly lower in the age 60 and over group than in other age groups [27].

Although a few reports show contrasting results [28-31], most of the available literature suggest that there is a rarefaction of cerebral arterioles and decrease in capillary density in aged animals and humans. However, there are still unresolved limitations with respect to measurement and quantitation. Immunohistochemistry (IHC), which is the most popular way to evaluate vascular morphology, needs to be improved. IHC results only show limited information in 2D planes and tissue processing has the potential to destroy or change vessel morphology unbeknownst to the researcher. A new technique introduced in 2013, termed CLARITY, is a method for chemically transforming intact biological tissues into a hydrogel-tissue hybrid [32]. This makes it possible to mark tissues with macromolecular labels such as antibodies while retaining original architecture and native biomolecular structure. However, CLARITY is still based on the traditional antigen-antibody reaction, as in IHC. Thus, neither IHC nor CLARITY can be used in the living brain. Moreover, existing methods for use in living mice including functional MRI (fMRI), two-photon microscopy (TPM) and wide-field optical microscopy have speed and resolution issues. Recently, a high-speed label-free functional photoacoustic microscopy method was reported. It was able to build 3D brain images with capillary-level resolution in living mice [33]. These emerging technologies and information are creating new opportunities for delving deeper into vascular aging research.

#### Arteriolar tortuosity

Arteriolar tortuosity consists of vascular spirals, loops and coils, often appearing in the white matter of the aged brain. Previous studies have revealed the association of tortuous cerebral vessels with age [34, 35].

**Figure 1.**
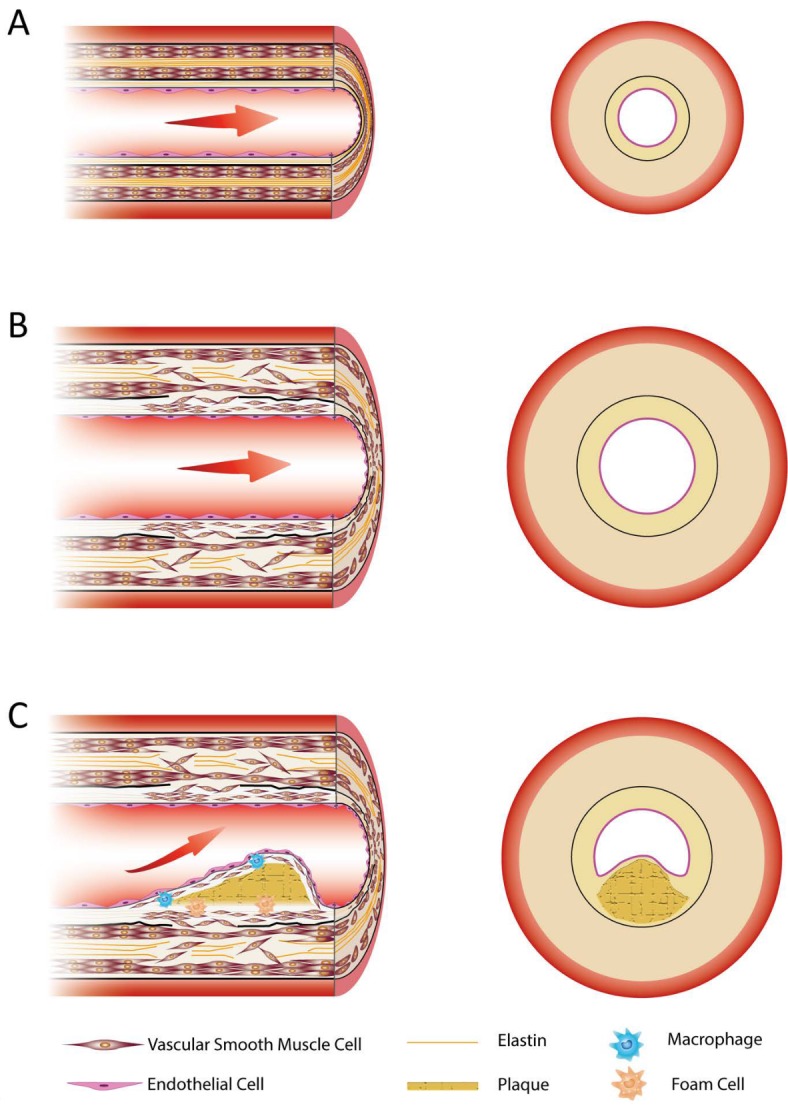
Illustrated histology of the normal vessel and histological alterations of vascular aging and atherosclerosis Cross sectional view of the arterial wall. (A) Normal artery. (B) Aged artery. It is characterized by a thickened vessel wall, thickened subendothelial layer, elastin fragments, VSMCs migration and invasion. (C) Atherosclerosis. It is characterized by the accumulation of plaque and the invasion of macrophages and foam cells.

Penetrating arterioles pass directly from the pial arteriolar network, remaining unbranched within the grey matter until its deeper layer. These arterioles enter into the white matter perpendicular to the brain surface. Spangler, et al. [36] counted the number of tortuous vessels and found it was increased with age, albeit not significant. Furthermore, they noticed that 70% of arteriolar tortuosity in the white matter was found at gray-white interfaces in the insular region and close to subcortical white matter in the inferior frontal and superior temporal gyri. Nonaka, et al. [37] also reported this phenomenon, but failed to confirm the age-related correlations by statistical measures. The number and tortuosity of vessels are low in the young; begin to increase in middle age and increase further in the aged human and mouse [27, 35, 38-41]. Microvascular tortuosity reduced blood flow and increased fluid shear stress, which become able to activate platelets and induce thrombosis [42-44]. Furthermore, mild chronic cerebral hypoperfusion can promote β-amyloid deposition in brain capillaries. Taken together, these findings suggest that tortuosity tends to advance with age, but is not an inevitable change that occurs in every individual. Although the association between arteriolar tortuosity and clinical disease remains obscure, it has been reported to be associated with hypertension [45], Alzheimer’s disease [46], and leukoaraiosis [34].

### 1.2 Microstructural remodeling in vascular aging

Vascular aging begins in young adults. As the arteries carry out the tasks of delivering energy and oxygen supply and directly participate in the development of CVDs, the majority of vascular aging studies focus on the artery, with only a few papers centering their attention on venous aging. Thus, in this section, we mainly review and discuss structural changes in arteries. However, this does not mean that venous aging cannot cause impairment. Venous dysfunction may be associated with acid-base balance, osmotic pressure balance and abnormal venous return that alter the brain microenvironment and possibly lead to cerebrovascular diseases or aggravate clinical symptoms and outcomes. Vascular aging causes remodeling of vascular structure, resulting in dysfunction of vessels and ultimately, cerebrovascular and cardiovascular diseases. Note that the vascular effects of aging per se should be distinguished from atherosclerosis, which is characterized by a variety of structural changes in the vessel wall including an accumulation of lipids in plaques (Fig. 1). Moreover, aging remodels the vascular wall more diffusely by largely different mechanisms compared to atherosclerosis. Mulvany, et al. [47] and van Varik, et al. [48] hypothesized that vascular remodeling can be classified macroscopically into different types due to the nature of the alterations occuring in the vessel.

Vascular structural remodeling can be “inward” or “outward” in relation to the lumen. At the same time, the vascular wall can be hypotrophic (thinned), eutrophic (invariant), or hypertrophic (thickened). Age-related change in elastic arteries is characterized histologically by a modest increase in diameter and gradual thickening of the vessel wall, resulting in outward hypertrophic remodeling while in muscular arteries and arterioles, aging is often manifested by eutrophic or hypertrophic remodeling. Whatever the type of structural remodeling, numerous mechanisms are involved.

In age-related structural remodeling, thickening of the vascular wall results from alterations in the intima and media [49], which are driven by multiple mechanisms. Studies on intimal-medial thickness (IMT) show progressive thickening during aging [50]. Some postmortem studies show that in human tissues, intimal-medial thickness is mainly the result of intimal thickening [49], whereas other studies found that normal vascular aging mainly produces medial thickening in the absence of atherosclerosis [51]. These differences are due at least in part to the examination of different regions of the artery. The mechanisms leading to the thickening of the vessel wall are vascular smooth muscle cell (VSMC) proliferation and migration, impaired integrity of elastin fibers and deposition of extracellular cell matrix (ECM).

Age-related vascular remodeling may differ from one species to another and vary in different vessels supplying blood to different organs. At the level of the aorta, the diameter of the aorta as well as the luminal diameter is enlarged gradually during aging. It was reported that the thickness of the media does not show a significant difference between aged and young subjects while the tunica intima thickens with age and results in the increasing aortic diameter [49, 52]. With advancing age, the endothelial cells lining the aortic lumen become irregular in shape. The thickening of intima occurs because of smooth muscle cells migrating from the media. The subendothelial space consists of collagens, proteoglycans and vascular smooth muscle cells. Intimal thickening contributes to reducing elasticity and the diameter of the lumen. Although the thickness of the media does not change significantly with age, its structure is altered. The aging process in the media is characterized by a decrease and fracturing of elastin fibers and by collagenous remodeling [53, 54]. At early ages, collagen fibers are almost absent in the aorta, but they begin to accumulate with advancing age [55]. In addition, the length of the aorta increases with age [56]. These age-related physiological alterations lead to a steady reduction in aortic elasticity and result in a less variable diameter between systole and diastole [57]. The thickening and stiffening of the aorta are linked to increased systolic blood pressure and pulse pressure.

At the level of the carotid artery, aging is a robust factor with respect to artery enlargement [58]. As in the case of the aorta, the thickness of the carotid intima increases with aging. However, progressive increases in medial thickness and lumen diameter are observed in both men and women, which is different from the case of the aorta [59]. In humans, the intima-media thickness of carotids from 90-year-old subjects is increased as much as two to three-fold, compared with that of 20-year-olds [60]. The thickening of the intima is due to the migration of VMSCs and an increase in extracellular subendothelial contents including collagens and proteoglycans. The increase in medial thickness is mainly due to SMC proliferation and increasing collagen content [61, 62] thus, contributing to age-related carotid stiffening. As in the aorta, the aging carotid media is characterized by a decline in elastin and increase in collagen. In general, the relative intimal thickening is greater than medial thickening. These changes increase diastolic diameter nearly linearly with age. Thus, the ratio of diastolic to systolic diameter and peak expansion velocity drop significantly in the aged [63].

At the level of muscular (in contrast to elastic e.g., aorta or carotid) arteries, there are relatively milder alterations with age. Muscular artery diameter increases with age and this increase is more significant in women [58, 64]. Consistent with the change in lumen diameter of aged carotids, the lumen diameter of muscular arteries increases in both men and women, although it is more pronounced in the latter [65, 66]. Both wall thickness and intima-media thickness are greater in aged subjects. Proliferation, hypertrophy and migration of VMSCs and accumulation of collagens and proteoglycans contribute to the increased intima-media thickness. However, the stiffness of muscular arteries remains relatively unchanged with age. This contradiction suggests that proliferation and hypertrophy of relatively compliant VMSCs offset the accumulation of components such as collagen. Therefore, although distensibility of the aorta and carotid declines with age in both men and women, distensibility and compliance of the muscular arteries seemingly remains unchanged [64]. This suggests that mechanical properties of muscular arteries contribute little, if at all, to age-related elevation of pulse pressure. Stiffening of elastic arteries with preservation of normal stiffness in muscular arteries gradually reduces the gradient of increasing arterial stiffness from the aorta to the periphery. In contrast to modest alterations in mechanical properties, endothelial function in muscular arteries deteriorates with age, which contributes to the age-related increase in pulse pressure [67].

At the arteriole level, increasing age is associated with a significant decrease in lumen diameter and an increase in media/lumen thickness ratio, with a tendency for the media-lumen ratio to be higher in women than men [68]. Arteriolar diameter increases with age [69] and the collagen to elastin ratio in arterioles is greater in elderly subjects [70]. Tortuosity often occurs in cerebral arterioles of aged subjects. Aging has a minimal effect on the mechanical properties or stiffness of arterioles. However, aging significantly impairs endothelium-dependent vasodilation [71, 72].

Capillary structure is rather different from that of arteries. Capillary luminal volume percentage and capillary density decrease with increasing age [73]. A sharp rise in apoptosis results in the loss and elongation of capillary endothelial cells [74]. Thickening of the basement membrane occurs with increasing age in both sexes [28, 75].

In the next section, we will discuss changes to the vascular cells and vessel walls from the intima to adventitia such as endothelial apoptosis and senescence, degeneration and changes in vascular elastin and collagen fiber.

#### Arterial intima

The intima has an average thickness of 50-100 μm with no significant difference between elastic and muscular arteries. Numerous studies show that the intima is altered dramatically with age in various species including rats, monkeys and humans [76-78].

##### Endothelial structure

Multiple molecular and cellular alterations take place in aged endothelial cells (ECs), which are likely to contribute to EC dysfunction. In the aorta, electron micrographs reveal ultrastructural changes of the endothelium in which end-to-end inter-EC junctions increase while overlapping or interdigital junctions (which are stronger and structurally more complicated) decrease, with aging. Immunostaining suggests that the expression of connexins CX37 and CX43 is gradually reduced with advancing age [79]. In carotid arteries, aging leads to the separation of endothelial cell-cell junctions, which increases permeability [80]. In the cerebral microcirculation, a series of reports demonstrated the loss of capillary ECs and elongation of the ECs with age [81-83]. Senescent cortical microvascular ECs show increased cell size and possess a polygonal shape. The cytoskeleton is reorganized prominently, with stress fibers accumulating at the cell periphery and with fewer focal adhesions. Aortic EC senescence produces similar cellular enlargement and cytoskeletal reorganization, but with decreased and shortened stress fibers and without changes in focal adhesions [84]. Aged human umbilical vein ECs (HUVECs) are characterized by increased cell size as well [85].

Levels of inflammatory mediators such as angiotensin II (Ang II), monocyte chemoattractant protein-1 (MCP-1) and milk fat globule EGF factor 8 protein (MFG-E8) are also increased with aging, which promotes the generation of reactive oxygen species (ROS) [86]. Inflammation, together with impaired telomere function, leads to a decrease in EC replicative capacity and increased susceptibility to apoptosis [87]. Furthermore, aged ECs show attenuated Jagged1 expression, resulting in enhanced VSMC proliferation, which is another key alteration involved in intima-media thickening and vascular dysfunction [88].

##### Endothelial apoptosis and senescence

In the aorta, coronary arteries, femoral arteries, capillaries and HUVECs, increased endothelial apoptosis is a feature of advanced aging and aging may also enhance the sensitivity of ECs to apoptotic stimuli [74, 89]. Apoptosis, an intrinsically activated form of programmed cell death, regulates cell elimination during vessel regression [90]. Asai, et al. [76] observed old monkeys and found that EC apoptosis increased while EC density decreased with age. This decrease accompanies impaired endothelium function. Therefore, endothelial apoptosis may reduce the number of healthy ECs, induce age-associated structural remodeling and promote EC dysfunction.

Complex molecular processes regulate apoptosis in ECs including numerous pro- and anti-apoptotic factors, membrane receptors and cysteinyl aspartate-specific proteases (caspases). Age-related upregulation of TNFα, calpain-1, MCP-1, ROS, MFG-E8 and caspase-9 and downregulation of endothelial nitric oxide synthase (eNOS) and SIRT1 activity increased sensitivity to apoptotic stimuli in ECs and promoted endothelial apoptosis [89, 91-93]. The activity of NF-κB, which is associated with endothelial apoptosis, is likely regulated by age-related nucleophosmin and the SIRT1 pathway [94-96].

Endothelial cellular senescence is another phenomenon that occurs in the aging endothelium. Normally, ECs rarely divide with a turnover rate of approximately once every three years [97]. As the proliferative capacity of ECs is limited, countless cells are in a state known as replicative senescence. These cells are unable to proliferate, although replicative senescent (RS) cells can maintain active metabolism for long periods of time [98]. Irreversible growth arrest may result in impaired regeneration and reduced EC number, contributing to endothelial dysfunction and impaired angiogenesis. One widely used marker of senescent cells is senescence-associated β-galactosidase (SA-β-gal). When its activity is measured at pH 6.0, β-galactosidase is expressed only in senescent cells. SA-β-gal activity is increased in ECs within atherosclerotic plaques and senescent HUVECs, but not in human coronary artery explants without signs of atherosclerosis [99]. As SA-β-gal staining can also be found in aged VSMCs, detected in the intima rather than the media, it cannot be used as a specific endothelial senescent marker. Senescence can be triggered by inflammation and oxidative stress, which can also cause telomere shortening [100-102].

Aging is associated with increased oxidative stress and oxidative damage [103]. The endothelium appears to be an important source of oxygen radicals [104]. Vascular ROS generation in rats substantially increases after mid-life. In young animals, upon ROS-induced activation, Nrf2 translocates to the nucleus where it binds to the ntioxidant responsive element (ARE) to activate transcription of antioxidant enzymes including NQO1, HO-1, GST and Trx. Thus, the Nrf2/ARE pathway is critical for endothelial protection in response to ROS [105, 106]. Age-related Nrf2 dysregulation contributes to vascular oxidative stress in aging. Even exogenous H_2_O_2_ treatment can neither activate Nrf2 nor induce the expression of the Nrf2-driven gene. Aging is associated with a downregulation of Nrf2 expression and upregulation of Keap1 expression, a cytosolic repressor, which can prevent nuclear translocation by interacting with Nrf2 in endothelial cells [107]. Induction of Nrf2-driven free radical detoxification pathways confers significant anti-apoptotic effects in cultured endothelial cells [108]. Thus, the rate of apoptosis in aortic segments under baseline conditions increases as a function of age. Age-related oxidative stress promotes vascular inflammation in aged arteries by activating the redox sensitive transcription factor NF-κB, which is consistent with the close correlation between vascular ROS production and expression of NF-κB target genes during vascular aging [109].

Endothelial progenitor cells (EPCs) also play an important role in endothelial maintenance by patching damaged regions and then differentiating into ECs [110]. EPCs serve as endogenous repair ‘workers’ to maintain the integrity of the endothelial monolayer by replacing denuded parts of the arterial intima, showing protective effects after both brain trauma and myocardial infarction [111, 112]. Advancing age was shown to be associated with reduced number and function of circulating EPCs, thereby enhancing vascular disease risk [113-115]. Edelberg, et al. [116] found that age-associated impairment of cardiac angiogenesis and vascular function can be restored by transplantation of bone-marrow-derived EPCs from young but not aged mice, suggesting that young and old EPCs may show different biology. EPCs recruited from transplanted young bone marrow cells could restore platelet-derived growth factor (PDGF)-mediated autocrine signaling while older EPCs lose the ability to express PDGF-B. Aging may also result in the shortening of telomere length in EPCs, thereby driving these cells into senescence. Improving the microenvironment of aged EPCs could rescue their impaired function. The injury repair capacity of aged EPCs is significantly greater when transplanted to young rats (compared with aged rats). Culturing aged EPCs in serum from young rats can rescue their decreased capacity to proliferate and migrate and increase expression of both phosphatidylinositol 3-kinase (PI3K) and eNOS, indicating that a young microenvironment can partially restore function in aged EPCs [117]. These results suggest that certain factors in the young serum may have potential treatment value. Mobilizing more circulating EPCs through the effects of food supplements has also been discussed as a possible approach in anti-aging medicine [118].

EPCs express high levels of antioxidant enzymes, e.g., superoxide dismutase and GPx-1 [119, 120]. GPx-1 KO mice have reduced blood flow recovery after ischemia compared with their wild type counterpart as EPCs require anti-oxidative enzymes for functional repair. EPCs derived from GPx-1 KO mice also show increased apoptotic sensitivity. Furthermore, EPCs treated with oxidants triggered apoptosis and decreased tube formation capacity [121]. Ang II is involved in the senescence and reduced proliferation of EPCs by promoting the expression of gp91phox and ultimately, O^2-^ formation. Although the detailed molecular mechanisms are not well studied, it is likely that age-related increased oxidative stress contributes to the dysfunction of EPCs [122]. Because ECs play important roles in the vascular endothelium and the molecular mechanisms of endothelial apoptosis and senescence have not been well defined, further study of endothelial aging is required for a better understanding of vascular aging.

##### Subendothelial alteration

Subendothelial thickness grows with increasing age. The number of VSMCs and the amount of collagen types I and III are increased in the intima of aged compared with young rodents [123]. Interestingly, inflammatory cells such as macrophages and lymphocytes do not infiltrate the thickened intima [124].

Wang, et al. [125] reported that the intima was thicker in 20-year-old than in 7-year-old non-human primates. Electron microscopy reveals infiltration by SMCs and deposition of matrix connective tissue in the intima beneath an intact endothelium without any evidence of atherosclerosis [76]. Consistent with findings in rodents, inflammatory cells were not found in the thickened intima. Similar changes in the aortic subendothelium can also be found in older humans, in the absence of lipid infiltration [78]. In the human brain, arteriolosclerosis is characterized by endocentrically-thickened arterioles characterized by VSMC infiltration and intimal deposition of collagen types I and III [126, 127]. Electron microscopic studies confirmed the accumulation of smooth muscle cells within the intima in human intracranial arteries [128]. These changes may contribute to vascular disorders and the increased risk of vascular diseases in the elderly.

#### Arterial media

##### Elastin aging

A series of studies showed that fracture of elastin fibers along with deposition of collagen can be considered characteristic of age-related arterial structural remodeling. The ratio of elastin to collagen in the vascular wall changes with increasing age, leading to many static and dynamic mechanical and biological effects. Elastin is closely associated with elasticity, which progressively deteriorates with age in the aorta and internal carotid artery of rats, resulting in their eventual stiffening [129-131].

In vertebral and basilar arteries of the cerebrovascular system in humans, aging increases arterial wall thickness and the ratio of collagen to elastin while reducing their distensibility [132]. Cross-sections of pial arterioles showed that elastin content was dramatically decreased in aged rats, whereas collagen content was not significantly changed [70]. Increased collagen and less frequently, elastin, were also seen in the media beneath large intimal plaques in human intracranial arteries [128].

Posterior circulation cranial arteries obtained from human cadavers revealed that the quantities of elastin remain constant in the old and young groups, consistent with similar results obtained from the thoracic aorta [133]. The functionality of deteriorated elastin is attributed to structural changes in the organization of elastin fibers. Elastin in the young is well organized circumferentially while in the old it is distributed heterogeneously throughout the wall without coherent orientation and is primarily seen in the inner media [134].

The reasons that drive elastin’s degradation are complex. An initial hypothesis assumed that elastin degradation was mainly because of material fatigue due to the cyclic stretching caused by pulsatile changes in blood pressure [134]. Consequently, factors such as hypertension and arrhythmia may accelerate this process as increased blood pressure converts into higher stress on load-bearing elastin fibers. However, further studies show many other molecular alterations related to elastin degradation. It thus seems that the degradation of elastin cannot be attributed solely to “material fatigue”. Recent studies in vascular aging have revealed a cell signaling pathway network involving Ang II and the activation of TGF-β1 and matrix metalloproteinases (MMPs) [123, 130]. These signaling pathways may contribute to the destruction of the elastin network and regulate collagen production by VSMCs.

In addition, the internal elastic lamina (IEL), a layer of elastic tissue, is located in the subendothelium, separating intima from media. It is believed that the media and adventitia of cerebral arteries contain relatively less elastin and more prominent IEL than the extracranial arteries [137]. The IEL was also reported to lose its structural integrity in the rat aorta [138]. In human posterior cerebral arteries, the aged IEL is composed of multiple fragmented layers while remaining thick and continuous. It is likely to be a stress-bearing structure at lower pressure, resulting in better compliance of small arteries, thus affecting vascular function in aging vessels.

##### Proliferation of VSMCs

In mice, rats and rabbits, aged VSMCs have an accelerated cell cycle when compared to their young counterparts [139-141]. For example, cultured VSMCs from old rat aortas show higher growth rates than those from young rats. Aged VSMCs show a higher percentage of S and G2 phases, with a lower percentage in the G0/G1 phase, compared with young VSMCs [142].

In aged VSMCs, cell cycle-associated proteins were found to be increased including cyclin D1, cyclin E, CDK2 and CDK4 and kinase activities associated with CDK2 and CDK4 [140]. Together with proliferative cell nuclear antigen (PCNA) and platelet-derived growth factor (PDGF) and its receptors, these proteins accelerate the cell cycle by facilitating the mitotic phase of VSMCs. The expressions of PCNA and CDK4 were increased by the elevated expression levels and interaction of MFG-E8 and integrin αVβ5 via ERK1/2 signaling [141]. MFG-E8, a regulator within the Ang II/MCP-1 VSMC signaling pathway, can not only enhance the VSMC proliferation, but also invasion and migration. Moreover, increased ROS production was reported in old mice VSMCs, leading to their high proliferative capacity [143]. The AP1 transcription factor gene, c-fos, was also over-expressed in aged VSMCs, which stimulates VSMC proliferation in old animals by interacting with the cyclin A promoter [144]. Emerging evidence suggests that ROS protect VSMCs from cell death and promote the proliferation of VSMCs [145, 146]. Inhibition of ROS could attenuate VSMC proliferation and inflammation by suppressing toll-like receptor 4, a well-known inflammatory-mediating receptor that have a critical role in initiating inflammation through increasing pro-inflammatory factor production.

However, the aging process may be much more complicated in human VSMCs. Proliferative capacity was increased with aging in VSMCs isolated from human tissues [78, 147], but contradictory studies report that aging leads to the loss of proliferative activity of human VSMCs [148, 149]. As mentioned above, the proliferation of VSMCs is, at least partially affected by the expression level of Jagged1 in ECs. It is still unclear to what extent the interaction of VSMCs with other vascular cells governs age-induced changes in structure or function since the most widely used *in vitro* cell culture methods employ single vascular cell types rather than a co-culture of different cell types [150]. Goubko and Cao [150] reviewed several methods for co-culture, which may be useful in vascular aging studies. Moreover, proliferation rate data from *in vitro* cell culture systems cannot simply be extrapolated for *in vivo* conditions for multiple reasons including the failure to recreate the particular microenvironment.

##### Intimal migration of VSMCs

Normal elastic fibers in young arteries are arranged in parallel elastic lamellae. Glycoproteins and integrins help VSMCs to anchor to the elastic lamellae [151]. These structures enable the vessel to bear the blood pressure produced by the heart and protect the vascular structure from overloading. Cell adhesion molecules of VSMCs detach while a dominant plasma membrane leading lamella, or leading edge, protrudes; this contacts an extracellular substrate and binds via transmembrane integrin receptors to form focal complexes and secure focal adhesions by actin polymerization [152]. Aged VSMCs also produce more MMPs to promote migration by detaching cells from the ECM [153] thus, inhibition of MMP activity suppresses VSMC migration in rats [154]. Moreover, degradation products of MMPs further damage deteriorating vessels [155]. The increased secretion of these ECM-degrading enzymes contributes to the loss of elastin and progressive fragmentation of the elastic lamellae.

The migration and invasion capacities of VSMCs increase with advancing age and Ang II signaling is known to be involved [123]. Exposure of cultured young VSMCs to numerous molecules including Ang II, TLR2, MFG-E8, PDGF-BB and MCP-1, significantly enhance their migration and invasion capabilities [123, 156-158]. Consistent with these results, the invasiveness of aged VSMCs can be blocked by the AT1 antagonist, losartan and the calpain-1 and MMP inhibitors. The intimal migration and invasion of VSMCs are also related to VSMCs proliferation and hyperplasia. PDGF-BB is known to generate intracellular ROS [159]. PDGF-BB-induced ROS could promote the proliferation of VSMCs and induce TLR4 expression. TLR4 and its associated pro-inflammatory cytokines have been shown to promote VSMCs proliferation and migration [146].

Taken together, the migration and invasion of VSMCs are key aging events that lead to diffuse intimal thickening, vascular stiffening and vascular dysfunction and may contribute to age-associated vascular diseases such as atherosclerosis.

#### Arterial adventitia

Serving as the vessel’s final line of defense, the adventitia is made up of elastic and collagen fiber bundles, fibroblasts, vasa vasorum, tertiary lymphoid organs and nerve structures. It takes part in regulating vascular tone and vascular nutrition supply [160]. Recent studies have indicated that with advancing age, T lymphocytes infiltrate the adventitia, which likely facilitates adventitia inflammation [161]. Other studies demonstrated that the adventitia is a site of local immune response in both aging and early atherosclerosis and promotes medial VSMC invasion into the intima [162].

Total collagen does not differ with age in adventitia. Collagen I and III deposition are observed in aged adventitia while elastin does not change. Fibroblasts in the adventitia synthesize more collagen than do VSMCs [163]. TGF-β1 expression in the adventitia increases with age, which enhances the expression of α-smooth muscle actin. Myofibroblastic differentiation and collagen synthesis are markers of the secretory phenotype of adventitial fibroblasts. Adventitial collagen fibers appear to be relatively wavy and ropy on scanning electron microscopy images of young subjects, but are significantly flattened and may have different mechanical properties in older patients [164]. As the adventitia participates in the regulation of vascular tone, the age-related increase in adventitial collagen deposition is accompanied by calcification, which contributes to arterial stiffness in aged subjects [165, 166].

Another characteristic of the adventitia is its niche-like properties, which resemble those of a stem/progenitor cell niche. The adventitia serves as a complex repository and a compartment where progenitor cells can interact with other constituents of the vessel wall. Loss of function of niche-dependent signaling and of progenitor cells is involved in early and late arterial wall disease and may be an important factor in age-related vascular remodeling [167]. However, more detailed work need to be done to investigate the natural fate of adventitial progenitor cells in aging arteries and whether depletion of the adventitial progenitor cell pool mediates age-related arterial dysfunction.

#### Capillary basement membrane thickening

Basement membranes (BMs) are extracellular matrixes that cover the basal side of endothelial and epithelial cells. They also surround fat, muscle and Schwann cells in multiple organs [168-174]. In the brain, cerebrovascular BMs are not only of importance in vascular development and formation and maintenance of the blood-brain barrier (BBB), but are also involved in the migration of peripheral cells such as leukocytes, into the brain [175-177]. BMs vary in different vessels. In cerebral capillaries, the BM is fused between ECs and the end-feet of astrocytes, forming a specialized perivascular ECM [178]. Together with capillary ECs, connected by tight junctions, astrocytic feet and pericytes, they form the exchange interface between the central nervous system and the blood stream.

The thickness of the BM increases in humans, rats and rhesus monkeys as they age [28, 29, 179, 180]. The BM thickness varies in different capillaries; even in a single capillary, BM thickness is not uniform [181]. Electron microscopy shows focal thickening of the BM, which can be more than twice as thick as an unaffected segment of the same vessel [28]. BM thickening may occur after reduced breakdown or increased synthesis of BM components such as collagen type IV, laminin, or heparin sulfate proteoglycans [29, 182]. As the BM is part of the BBB, pathological changes in the aging BM may affect BBB function and could contribute to age-related cognitive impairment and Alzheimer’s or other neurodegenerative diseases [180, 183].

## 2. Age-related vascular dysfunctions

The structural effects of aging lead ultimately to vascular dysfunction, which can be attributed to vascular stiffness, impaired endothelial function and increased BBB permeability.

### 2.1 Arterial stiffness

Arterial walls stiffen with advancing age, which reduces their mechanical elastic properties (arteriosclerosis). A Framingham Heart Study offspring cohort consisting 188 men and 333 women was conducted to assess carotid-femoral (central) and carotid-brachial (peripheral) pulse wave velocity. These volunteers were free of clinical cardiovascular diseases, hypertension, diabetes, smoking within the past 12 months, dyslipidemia, or obesity. Central arterial stiffness was noted to progress with advancing age with little change in peripheral arterial stiffness [184]. Another large landmark study, the Baltimore Longitudinal Study on Aging, confirmed the increasing central arterial stiffness with age by carotid-femoral pulse wave (cfPWV); the cohort consisted of 354 men and 423 women who were 21 to 94 years old and free of clinical cardiovascular diseases [185, 186]. Studies with large cohorts of 4659 and 1489 volunteers from China and Korea, respectively, reported the age-related progression of arterial stiffness as well. Cardio-ankle vascular index was used in these reports, which is a non-invasive method and is independent of blood pressure [187, 188]. Large elastic arteries exhibit significantly more stiffness than peripheral and muscular arteries [187, 188]. In the young, large arteries are more elastic with a reflected pressure wave arriving centrally during diastole, which does not increase cardiac afterload. Pulse pressure is substantially higher in the periphery as compared with the central artery [189]. In middle-aged individuals, increasing pulse wave velocity leads to earlier return of the reflected pressure wave to the central aorta during systole, which augments central systolic and pulse pressure and reduces peripheral amplification [190]. Finally, in the elderly, increasing central arterial stiffness exceeds peripheral arterial stiffness to result in the earlier return of pulse waves at end-systole rather than during diastole, which has the potential to double the afterload on the left ventricle [191]. Loss of the arterial stiffness gradient with aging may increase transmission of a larger potentially harmful pressure wave into the microcirculation and lead to hypoperfusion in the microcirculation.

Peripheral blood pressure increases with age. Similarly, loss of the normal arterial stiffness gradient reduces pressure amplification (peripheral/central pulse pressure ratio), which has prognostic significance for cardiovascular risk and may damage the microcirculation [192].

In contrast to peripheral muscular arteries, cerebral arteries become dysfunctional with age even though their elastin content is not reduced. Instead, aging leads to fragmentation of elastin. Vascular integrity becomes compromised with the reduction in the number of VSMCs, atrophy of cerebral arterioles and declining distensibility [134]. This process may be implicated in hippocampal sclerosis of aging and other neurodegenerative conditions related to small vessel disease [70, 126].

Aging leads to morphological and functional changes at all levels of the vascular tree. In general, the relative amounts and the ratio of elastin and collagen are linked with arterial stiffness. Proximal parts of the arteries are more elastic, consisting a relatively larger proportion of elastin than collagen, when compared with distal vessels. Collagen accumulation and elastin degeneration and fragmentation occur in the aged arterial wall, together with endothelial dysfunction and thickening, promoting vascular stiffening [186]. The increased accumulation of MMPs in the tunica media and the activation of inflammation destroy vascular structure. In addition, aging induces differentiation from the normal contractile type to proliferative, synthetic or migratory phenotypes of VSMCs.

Vascular stiffness in aged adults may have a heritable component [193]. A genome-wide association study reported a single-nucleotide polymorphism, rs3742207, in the COL4A1 gene on chromosome 13, which is related to pulse wave velocity (PWV) [194]. Two twin studies from the United States and Italy showed that AIx aortic augmentation index (which measures the central impact of pulse-wave reflection) and aortic PWV are moderately heritable with significant intrafamilial concordance [195-197].

Stiffening is the basic functional impairment underlying vascular aging. Overall, it is related to increased systolic blood pressure, pulse pressure, pulse wave velocity and decreased diastolic pressure; it is thought to contribute to vascular insults detected by MRI, brain aging and memory deficits [184, 198-202].

### 2.2 Endothelial dysfunction

The endothelium is one of the largest endocrine organs in the human body. Vascular ECs secrete a series of biologically vasoactive molecules (through both autocrine and paracrine processes) that play important roles in maintaining vascular structural and functional stability. Healthy endothelium maintains vascular structure and regulates vascular tone, which has the potential to directly lower vascular resistance. Aging is an independent impact factor for endothelial dysfunction even without clinical cardiovascular disease or major risk factors [203]. Several clinical studies have shown that endothelium-dependent vasodilatation progressively declines with age. This observation has been consistently seen in coronary arteries [204], brachial artery [205, 206] and the peripheral arteries [207]; it occurs earlier in men than in women [205]. These observations in humans are supported by many animal studies performed in rats [208], rabbits [209], and mice [210]. The presence of endothelial dysfunction in old people is not only associated with cardiovascular disorders [211], but also with diseases related to aging such as erectile dysfunction [212]. renal dysfunction [213] and retinopathy [214].

The pathophysiological mechanisms of age-dependent endothelial dysfunction are likely multifactorial. In addition to impaired endothelium-dependent vasodilatation, these include the imbalance between vasodilation and vasoconstriction, growth promotion and growth suppression, thrombosis and anti-thrombosis, inflammation and anti-inflammation_._

The balance between vasodilation and vasoconstriction can be modulated by the synthesis and release of vasodilators (e.g., NO, prostacyclin and endothelium-derived hyperpolarizing factor) and vasoconstrictors (e.g., endothelin-1, Ang II and thromboxane A_2_). Growth of vessels can be controlled by the balance between pro-angiogenic (e.g., vascular endothelial growth factor-mediated) and anti-angiogenic (e.g., endostatin-mediated) signaling [215]. Inflammation, together with oxidative stress, are considered to be the main mechanism of age-associated endothelial dysfunction. Cytokines that can promote and reflect vascular inflammation include TNF-α, interleukin-1 beta (IL-1β), members of the super family of interleukin 6 (IL-6) and C-reactive protein (CRP) [216]. Another mechanism, involving ROS, not only participates in regulating cell growth and adaptive responses, but also may induce cellular injury and death at higher concentrations [217]. Other basic endothelial processes including apoptosis, repair and regeneration are likewise associated with ROS signaling [217]. Thrombosis is critical in the pathogenesis of cardiovascular and cerebrovascular diseases. Thrombosis and anti-thrombosis are affected by the endothelium and its function, although arterial thrombus formation is seemingly not aggravated by age-dependent vascular dysfunction in the absence of additional risk factors, at least in mice [218]. Further studies on human subjects need to be conducted to confirm this finding, although evaluating the direct effects of aging on human vessels is difficult because other risk factors (e.g., atherosclerosis, hypertension, diabetes) need to be taken into account. Nevertheless, expression and activation of cytokines and other mediators change with age and may lead to impaired vascular homeostasis and endothelial dysfunction even in the absence of such confounding factors [216].

Loss of normal endothelial function is the initial stage of pathogenesis triggering atherosclerosis. Cardiovascular diseases are strongly related to endothelial dysfunction [219] with more cardiovascular events occurring in hypertensive patients with higher grades of endothelial dysfunction [220]. Cerebral endothelial dysfunction allows substances from the blood to enter the vessel wall, causing thickening and structural disintegration, perivascular brain damage, and neurological disease. Endothelial dysfunction is a key antecedent of clinical vascular disease and may serve as a predictive and potential diagnostic marker of disease [221, 222]. Given its essential role in coronary, cerebrovascular and peripheral arterial diseases, approaches focused on preserving or improving vascular endothelial dysfunction may have a role in alleviating age-related disease.

### 2.3 Hypoperfusion

Advancing age is associated with structural and functional alterations of blood vessel walls, resulting in increased vascular stiffness and endothelial dysfunction. As a high flow and low impedance organ, the brain microvasculature is constantly exposed to pulsatile hemodynamic strain. Arterial stiffening and the resulting increase in pulse pressure could lead to elevated cerebrovascular resistance and ultimately, hypoperfusion. Such arterial stiffening with aging is negatively associated with cerebral blood flow and the cerebrovascular conductance index [223]. In human aging studies, the cerebral perfusion of healthy volunteers was assessed in different age groups. Total CBF decreases with advancing age while systolic and pulsatile CBF increase, which is independently associated with central arterial stiffness [224]. Decreased blood flow velocities and a concomitant increase in pulsatility occur in the middle, anterior and posterior cerebral arteries with advancing age, especially in those ages 40 and above [225, 226]. Aging-associated cortical CBF reduction is greatest later in life (after age 60), rather than it being a linear process [227]. The left hemisphere was associated with a greater number of regions with reduced CBF than the right hemisphere, specifically the lateral occipital and supramarginal regions [228]. The CBF declines in the frontal, temporal and parietal regions while subcortical CBF was relatively preserved with age [227, 229-231]. These data show that both the regional cerebral perfusion rate and the flow velocity in the cerebral resistance vessels decrease in healthy aging humans. Across studies, the most consistent finding in normal aging is decreased frontal lobe metabolism while the temporal, parietal, and occipital lobes’ metabolism vary considerably among volunteers [232-234]. Normal aging mostly affects medial frontal brain regions while AD-related alteration is mostly observed in the lateral frontal cortex [235]. Oxygen and glucose are the primary components for the generation of energy in neurons. Cerebral hypoperfusion can cause a critical ATP depletion, promoting mitochondrial dysfunction. Meanwhile, mitochondrial dysfunction leads to increased ROS production [236]. The imbalance of ROS and antioxidants results in oxidative damage, which impacts vascular tone and in turn aggravate the cerebral hypoperfusion [237]. Furthermore, the rise of ROS signaling leads to BBB breakdown. EC integrity is disrupted by increasing oxidative stress [238]. Oxidative stress is in turn able to activate MMPs. Free radicals stimulate the degradation activity of MMPs; this can occur at the tight junctions and therefore disrupt the BBB. BBB breakdown due to oxidative stress-induced MMP activation is also a mechanism of cerebral ischemia-reperfusion injury [239]. Besides, astrocytes, which constitute the major ultrastructural change in the BBB, generate ultrastructural abnormality as a result of chronic cerebral hypoperfusion. Astrocytic foot processes vacuolization is the extension of the degeneration of the astrocytic body. It is suggested that the BBB is prone to structural weakness and functional instability [240]. Hypoperfusion is also aggravated by the ischemia-induced pericytes death, which increases resistance to microvascular flow and produces a long-lasting decrease of capillary blood flow as well as a breakdown of the BBB [240]. All in all, these effects could continue spiraling downward, which could aggravate regional CBF reduction (and reduction of oxygen/glucose supply to the brain), to ultimately accelerate the neurodegenerative process.

### 2.4 Blood-brain barrier

The BBB acts as a diffusion barrier and is composed of capillary ECs, basal lamina and astrocytes as well as tight junctions between the ECs. The normal BBB plays a key role in regulating the entry of solutes and ions and the migration of immune cells into the central nervous system. It thereby regulates the environment of the normal neuron and glial cell [241]. Normal aging appears to have an independent effect on BBB structure and function. At the BBB, these changes include a decrease in cortical and white matter microvascular density. Extracellular matrix components accumulate in the vascular basement membrane, leading to the thickening of the basement membrane. Disruption of the tight junction complex occurs by decreased expression of TJ proteins such as occludin and zonula occludens 1 [242]. The vessel wall stiffening and endothelial dysfunction lead to high pulsatility and hypoperfusion of the brain and the degeneration of the astrocytic body occurs. It is suggested that the BBB is prone to structural weakness and functional instability [240]. For many years, BBB permeability studies reported different findings ranging from none [243, 244] to little significant changes in permeability [245]. Differences in animal models and assessment techniques might account for some of these differences. Thus, in 2009, Farrall and Wardlaw [246] reviewed studies on BBB permeability in living humans and published a meta-analysis, which revealed that increasing age was associated with increased BBB permeability. Furthermore, BBB permeability was further increased in patients with either vascular or Alzheimer’s dementia compared with age-matched controls. A recent brain imaging study (MRI) confirmed these findings. BBB breakdown during normal aging occurs initially in the hippocampus, a region critical for learning and memory. The BBB breakdown is more significant in mildly cognitive impaired individuals compared with age-matched controls, indicating a possibility that the breakdown of the BBB may contribute to early cognitive impairment [247].

Rodent models show structural alterations of the BBB manifested by leakage of endogenous albumin and IgG [248, 249]. The permeability of the BBB was increased in the perivascular area and the hippocampus of aged *vs.* young rats [250]. BBB function was also impaired with advancing age in SAMP8 mice, a senescence-accelerated mouse strain that shows increased oxidative stress and age-associated pathological phenotypes at an early age [251]. Hyper-permeability of the BBB with microglial activation was found in aged Wistar rats [252]. Such microglial activation may be triggered by the entry of molecules such as lipopolysaccharides, across the BBB. In turn, microglial activation is a source of oxidative free radicals, which can generate a vicious cycle that damages the BBB further. Thus, alterations of BBB function are strongly associated with oxidative stress in the brain. TNF-α transport is also increased in the occipital cortex, midbrain and striatum of aged SAMP8 mice, impairing learning and memory [253]. Furthermore, the BBB permeability of ovariectomized female rats is increased two- to four-fold compared with their young controls. Decreased estrogen levels may participate in age-related BBB dysfunction and associated neurodegeneration [254]. Although the BBB has a relatively higher permeability in reproductive senescent Sprague Dawley rats, estrogen replacement treatment increases BBB leakage to two- to four-fold in the olfactory bulb and hippocampus compared with young ovariectomized rats [254], which indicate that estrogen replacement therapy differentially regulates BBB permeability in young and reproductive senescent female rats.

Therefore, understanding the effects on BBB permeability caused by the aging vasculature could provide insights into possible mechanisms of vessel wall alterations and associated brain parenchymal damages in neurologic disease.

## 3. Summary

Aging modifies the structures of blood vessels even down to the level of the capillary basement membrane. These structural alterations can then lead to vascular stiffness, impaired endothelial function and increased BBB permeability. A thorough study of the underlying mechanisms and key mediators of these age-related vascular dysfunctions would be beneficial in the search for potential interventions.
